# The Role of Metals in the Reaction Catalyzed by Metal-Ion-Independent Bacillary RNase

**DOI:** 10.1155/2016/4121960

**Published:** 2016-12-21

**Authors:** Yulia Sokurenko, Vera Ulyanova, Pavel Zelenikhin, Alexey Kolpakov, Dmitriy Blokhin, Dieter Müller, Vladimir Klochkov, Olga Ilinskaya

**Affiliations:** ^1^Institute of Fundamental Medicine and Biology, Kazan Federal University, Kremlevskaya Str. 18, Kazan 420008, Russia; ^2^Institute of Physics, Kazan Federal University, Kremlevskaya Str. 16a, Kazan 420008, Russia; ^3^Institute for Anatomy and Cell Biology, Justus Liebig University Giessen, Aulweg 123, 35385 Giessen, Germany

## Abstract

Extracellular enzymes of intestinal microbiota are the key agents that affect functional activity of the body as they directly interact with epithelial and immune cells. Several species of the* Bacillus* genus, like* Bacillus pumilus*, a common producer of extracellular RNase binase, can populate the intestinal microbiome as a colonizing organism. Without involving metal ions as cofactors, binase depolymerizes RNA by cleaving the 3′,5′-phosphodiester bond and generates 2′,3′-cyclic guanosine phosphates in the first stage of a catalytic reaction. Maintained in the reaction mixture for more than one hour, such messengers can affect the human intestinal microflora and the human body. In the present study, we found that the rate of 2′,3′-cGMP was growing in the presence of transition metals that stabilized the RNA structure. At the same time, transition metal ions only marginally reduced the amount of 2′,3′-cGMP, blocking binase recognition sites of guanine at N7 of nucleophilic purine bases.

## 1. Introduction

The T1 family (EC 3.1.27.3) ribonucleases (RNases) hydrolize RNA and cleave the 3′,5′-phosphodiester bond between guanosine 3′-phosphate and the 5′-OH group of the adjacent nucleotide, forming a 2′,3′-cyclic guanosine phosphate in the first stage of a catalytic reaction. This stage is reversible and is much faster than the second, in which the cyclic intermediate is hydrolyzed to a corresponding 3′-phosphate [1]. Unlike pyrimidine specific RNases that belong to the family of RNase A (EC 3.1.27.5), RNases T1 are guanyl specific; hence, 2′,3′-cyclic guanosine monophosphate (2′,3′-сGMP) can only be cleaved in the second step of catalysis [1]. Many members of genus* Bacillus* secrete RNases that can perform both stages of the catalytic reaction. Specifically, such species are* B. pumilus* and* B. licheniformis* that can be isolated from the human gastrointestinal tract, representing resident rather than transient microbiota [2]. The amount of such microorganisms in the gastrointestinal tract significantly exceeds that which can be expected to come from food. Germination of* Bacillus* spores in the human intestine and transient colonization are part of the life cycle of human related bacilli [3]. Earlier, we have found* B. pumilus* in the biopsies of human rectal epithelium after preoperative bowel cleansing [4]. Hence, products that are secreted by bacilli can affect the human normal flora and mediate changes in functional activity of the human body. Extracellular bacterial enzymes are key agents in relation to the human body, as they directly interact with epithelial and immune cells [5]. However, it would be erroneous to exclude from this interaction the reaction intermediates, such as 2′,3′-cyclic positional isomers of classic second messengers, namely 3′,5′-cAMP and 3′,5′-cGMP. For instance, it is shown that 2′,3′-cyclic nucleotides are involved in the regulation of mitochondrial permeability [6, 7]. In addition, 2′,3′-cGMP can promote thymidine incorporation in the DNA of lymphocytes [8] and, similar to 3′,5′-cGMP, can increase severalfold cGMP-dependent ATPase [9]. Although the biological role of 2′,3′-cGMP has not yet been studied in detail, it is clear that noncanonical cyclic second messengers, such as 2′,3′-cGMP and 2′,3′-cAMP are produced by animals and plants in response to stress situation that induces RNA degradation [10, 11].

It is known that cyclic phosphates can only be hydrolyzed by RNase after all poly- and oligoribonucleotides have been cleaved [12]. Recently, we have established that 2′,3′-cGMP can be maintained in the RNA:binase reaction mixture for more than one hour, which enables binase to manifest its biological effects [13]. Although binase is treated as an enzyme which does not require metal ions for RNA catalysis [14], the objective of this study was to investigate whether divalent metal ions can affect the ability of binase to form 2′,3′-cGMP cyclic intermediate.

## 2. Materials and Methods

### 2.1. Materials

We used guanyl preferring RNase, binase (molecular weight 12.3 kDa, 109 amino acid residues, pI = 9.5), isolated as a homogeneous protein with catalytic activity, from a culture fluid of a recombinant strain of* Escherichia coli* BL21, carrying pGEMGX1/ent/Bi plasmid. Binase catalytic activity toward yeast RNA is 14000000 U/mg at pH 8.5 [15].

Positional isomers of cyclic nucleotides 3′,5′-cGMP and 2′,3′-cGMP, tRNA from* Torula* yeast, and 3′,5′-cGMP were purchased from Sigma-Aldrich (Germany). Ions of transition (Mn^2+^, Fe^2+^, and Co^2+^) and nontransition (Mg^2+^, Ca^2+^, and Zn^2+^) elements were compounded in the reaction mixture in the form of high-purity chlorides (analytical grade).

### 2.2. Determination of Binase Cyclizing Activity

Binase was incubated in a total volume of 200 mcL in 0.5 M Tris-HCl buffer (рH 8.5) with yeast RNA at concentrations, defined for specific experiments, at 37°C for 15 min. The catalytic reaction was stopped in cold. Then, 100 mcL EPBS (8.24 мМ Na_2_HPO_4_, 1.77 мМ NaH_2_PO_4_, 140 мМ NaCl, and pH 7.4) was added, and the solution was thoroughly mixed and centrifuged for 2 min at 12000 ×g. The supernatant was decanted into a tube containing 60 mcL EPBS and frozen at −80°C. The obtained 2′,3′-cGMP product was determined by enzyme immunoassay (ELISA), using antibodies against 3′,5′-cGMP.

### 2.3. ELISA Measurement of 2′,3′-cGMP Quantities

We used commercial 96-well plates with immobilized goat anti-rabbit antibodies (secondary antibody), primary rabbit anti-3′,5′-cGMP, and the appropriate reagents in accordance with manufacturer's instructions (IHF, Hamburg, Germany). RNA hydrolysis by guanyl-specific microbial RNases does not lead to the formation of 3′,5′-cGMP [14]. Earlier, we have shown that 2′,3′-cGMP can be detected in cross-reaction with commercial 3′,5′-cGMP antibodies for 2′,3′-cGMP concentrations that exceed 10^−5^ М (10 nM/mL and higher) [13]. In total, 50 mcL of test sample, 50 mcL of biotin solution, and 100 mcL of rabbit primary 3′,5′-cGMP antibodies were added to the well and incubated overnight at 4°C. Then, 200 mcL of freshly prepared solution of streptavidin-horseradish peroxidase conjugate was added to the well, which was incubated for 30 min at 4°C. Thereafter, the plate was washed three times with buffer, filled with 250 mcL of 3′,3,5′,5-tetramethylbenzidine solution, and maintained at 4°C for 40 min. Then, 50 mcL of 2 M H_2_SO_4_ was added and the plate was incubated for 5 min and analyzed on a colorimeter at 450 nm. The colour intensity is inversely proportional to the amount of cyclic nucleotides that bound to antibody. Concentrations of cyclic nucleotides were calculated by using the calibration curve [13].

### 2.4. NMR Spectroscopy

Binase ^1^Н NMR-spectra in an aqueous solution that contained chlorides of transition (Mn^2+^) and nontransition metals (Mg^2+^, Zn^2+^), as well as tRNA that contained Mn^2+^ in aqueous solution, were recorded on a NMR spectrometer AVANCE III-700 (700 MHz, Bruker, Germany), equipped with a quad cryogenic sensor (^1^H, ^13^C, ^15^N, and ^31^P) CryoProbe. ^1^Н NMR recording of tRNA spectra in the aqueous solution with ions of nontransition metals (Mg^2+^, Ca^2+^, and Zn^2+^) was performed by using an NMR spectrometer AVANCE II-500 (500 MHz, Bruker, Germany).

The spectrometers operated in the mode of internal stabilization at the resonance line ^2^H. The ^1^H-NMR-spectra were recorded using 90° pulses (pulse relaxation delay 2 с; spectral width 20.00 ppm; the number of accumulations of 100). NMR-spectra were recorded at 298 K, pH 5.5.

## 3. Results

### 3.1. Spectral Characteristics of RNase and RNA's Interaction with Divalent Metal Ions

**Figure 1:**
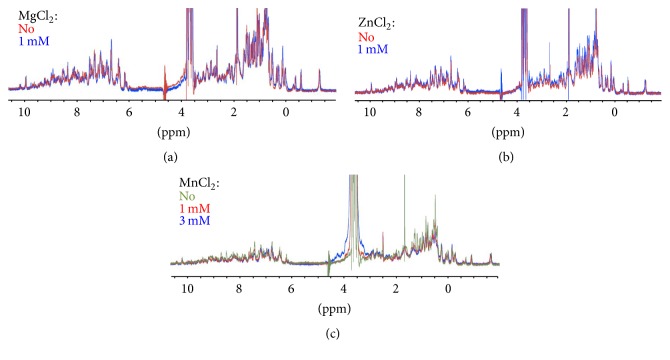
Regions of ^1^H NMR titration (700 MHz) for binase with increasing amount of (a) MgCl_2_ from 0 to 1 mM, (b) ZnCl_2_ from 0 to 1 mM, and (c) MnCl_2_ from 0 to 3 mM. Binase concentration was 1 mg/mL. NMR-spectra were recorded at 298 K, pH 5.5.

**Figure 2:**
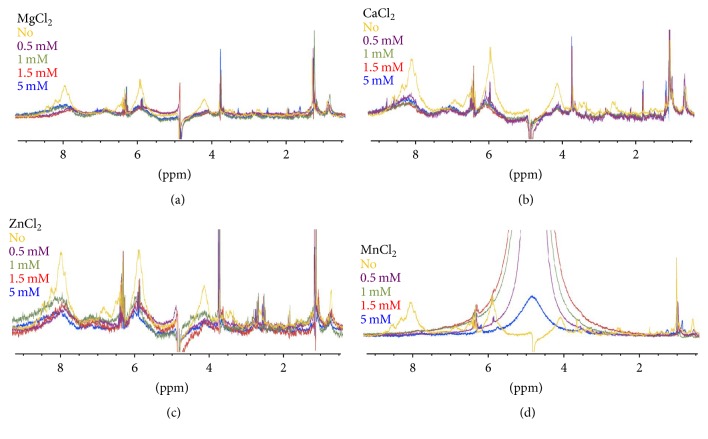
Regions of ^1^H NMR titration (500 MHz) for RNA with increasing amount of (a) MgCl_2_, (b) СаCl_2_, (c) ZnCl_2_, and (d) MnCl_2_ from 0 to 5 mM. RNA concentration was 1 mg/mL. NMR-spectra were recorded at 298 K, pH 5.5.

As binase activity is not dependent on any cofactors or metal ions [14], the spectral characteristics of binase were not changed when divalent ions were added to the protein (Figure 1). RNA interaction with Mn^2+^ led to a broader signal in the NMR spectrum (Figure 2(d)). Therefore, it can be assumed that site-specific Mg^2+^, Ca^2+^, and Zn^2+^-RNA interactions are not present in RNA. The relation of the NMR spectral width and the amount of salt in the studied concentrations (0.5–5 mМ) was found to be insignificant. Unlike Mn^2+^, these ions do not have strong paramagnetic properties and, therefore, have no pronounced effect on RNA in the NMR spectrum (Figures 2(a), 2(b), and 2(c)). Speaking of RNA-Mn^2+^ interaction, we could observe a selective and progressive broadening of NMR signal lines in the ^1^H NMR spectrum of tRNA in the region of 5–8 ppm where the proton signals of nitrogenous bases are located (Figure 2(d)). The NMR signal lines disappeared after we increased the concentration of MnCl_2_. Due to a significant decrease in the relaxation time for protons in the vicinity of the paramagnetic center, we detected nucleotides that had nonzero dipole-scalar interaction with electron spin of metal's nucleus. The recorded shorter relaxation time, as well as similar changes in the NMR-spectra that are associated with the interaction of Mn^2+^ and Co^2+^ with histidine residues and peptide bound glutamate can be due to Mn^2+^ activity [16].

### 3.2. Change in the Rate of 2′,3′-cGMP in RNA Catalytic Cleavage by Binase due to Divalent Metal Ion Activity

**Figure 3:**
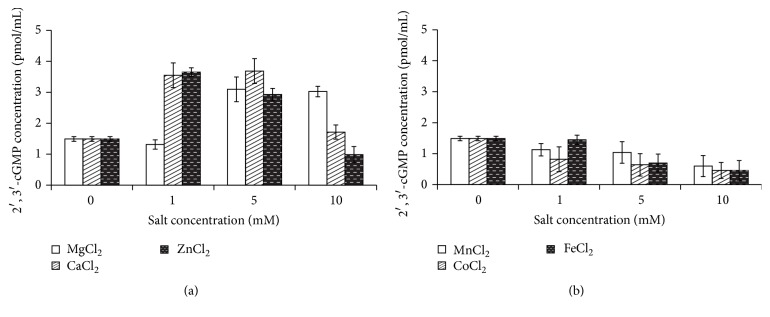
The impact of nontransition (а) and transition (b) metal ions on the rate of 2′,3′-cGMP during catalytic cleavage of RNA by binase.

Using antibodies that are applied to define the rate of the intermediate metabolite 2′,3′-cGMP in the tRNA-binase reaction, we have shown that the level of the 2′,3′-cGMP product increased by 2–2.5 times in a reaction mixture containing nontransition metal ions compared to the mixture that had no ions added (Figure 3(a)). Mn^2+^, Co^2+^, and Fe^2+^ had no stimulating impact on the rate of 2′,3′-cGMP. On the contrary, the rate of 2′,3′-cGMP insignificantly dropped due to higher levels of ion concentration (Figure 3(b)).

## 4. Discussion

It is known that tertiary RNA structure can be stabilized by divalent metal ions at physiological pH [17]. Previously it was thought that since RNA can renature in the presence of both divalent and monovalent ions, divalent ion binding to RNA is due to a common electrostatic interaction of ions with phosphates [18]. Later it was shown that the associative behaviour of metal cations towards RNA depends on its structural organization and nucleotide sequence [19]. In addition to formation of complexes with negatively charged deprotonated phosphates, metal cations also interact with some electron donors of oxygen and nitrogen atoms that are part of nitrogenous bases, excluding exocyclic nitrogen atoms [17]. The interaction is due to both stable binding of RNA by fully solvated cations via one or two layers of water molecules and strong relationships of negatively charged functional RNA groups, which are directly coordinated by partially dehydrated cations [20]. Today it is clear that divalent metal ions play a key role in the allosteric regulation of enzymatic activity of RNA [21, 22].

It is difficult to interpret the role of ions, as it is hard to distinguish between the direct catalytic impact of ions and the indirect role of ions in active RNA structure. Recently, it has been found that the transition metals such as Ni^2+^, Co^2+^, and Mn^2+^ have specific binding sites in RNA, in particular in the riboswitch structure [23]. Mg^2+^, Mn^2+^, and Zn^2+^ interact with guanosine as a cosubstrate and change the parameters of the catalytic reaction in the first step of splicing [24]. Ions of transition and nontransition metals are not interchangeable in terms of manifested regulatory effects. For instance, riboswitches that are found in the 5′-untranslated region of the* Salmonella typhimurium* transcription, which is conserved among Gram-negative enteric bacteria, are allosterically activated by Mn^2+^, rather than by Са^2+^ or Мg^2+^ [25]. For several group I intron ribozymes, folding and catalysis activate the ions of Mg^2+^, but not Mn^2+^. It was established that, for intron of 23S rRNA gene of the large ribosomal subunit* Chlamydomonas reinhardtii*, Mn^2+^ has no inhibitory effect on catalysis but causes misfolding of ribozyme. GMP, a cosubstrate of the group I introns displaces Mn^2+^ from the binding site of guanosine (GUC) and restores the folding [26].

We have shown that transition and nontransition ions have different impact on the tRNA structure and 2′,3′-cGMP (Figures 2 and 3). tRNA consists of 3-4 strong (Kd ≈ *μ*M) and more than 20 weak Mg^2+^-binding sites [27]. The latter includes the 8–12 turn (Op-U8, Op-A9), D-loop (Op-A20, Op-A21), D/TΨC-loop-1 (Op-G19, N7-G20), D/TΨC-loop 2 (Op-G57, Op-A14), acceptor arm (G3•U70), and the anticodon loop (Op-Y37) [28]. RNA-Mg^2+^ interaction is primarily mediated by direct binding of oxygen atoms in spatially close phosphate groups and, to a lesser extent, by binding of 2′-hydroxyl groups, N7 atom of purine bases, and keto-oxygen atoms (U and G). RNA-Mg^2+^ binding motifs are represented by (1) the major groove of mixed G•U pair, followed by Y•G pair; (2) shifted G•A pair; (3) magnesium “clamp”; (4) metal ion lightning; (5) E-loop motif; (6) AA platform; and (7) phosphate guanine binding motif [29].

**Figure 4:**
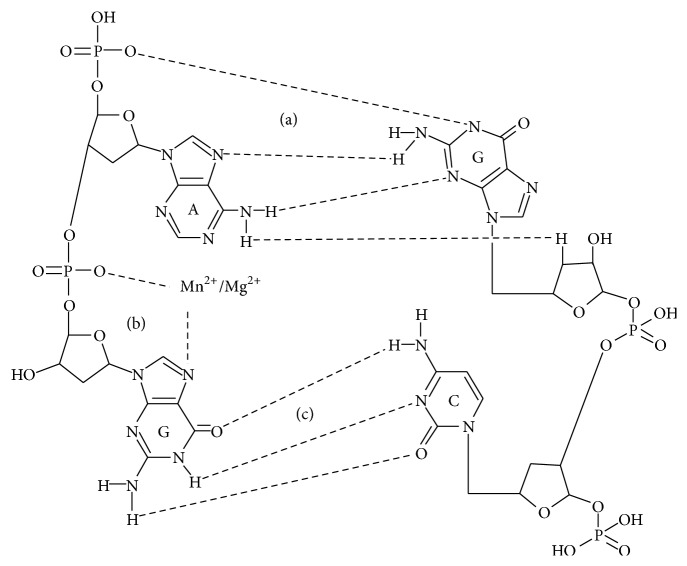
Binding site of Mn^2+^ and Mg^2+^ ions in RNA. Noncanonical (G•A) (а) and canonical Watson-Crick (G:C) (c) binding of RNA nucleotides. Thermodynamic stability of G•A and G:C (b) pairs is due to four and three hydrogen bonds, respectively. Hydrated ions of Mn^2+^ or Mg^2+^ (water molecules that are involved in coordination of RNA by metal ions are not depicted in the figure) coordinated the N7 level of guanine for the G:C pair and oxygen phosphoric acid residue of the adjacent phosphodiester bond; Mn^2+^ has a greater affinity to nucleophilic nitrogenous bases, and Mg^2+^ tends to form a rigid bond of P–O.

Unlike Mg^2+^, Mn^2+^ ions are only bound in the tRNA molecules with the D/TΨC-loop-1 and the acceptor arm [28]. Only one of the seven motifs that are characteristic of Mg^2+^-binding sites, namely, the shifted G•A pair, is reported for Mn^2+^ (Figure 4) [29].

Despite having similar radius, charge, and RNA site, Mg^2+^ and Mn^2+^ are not identical [30]. Mg^2+^ and Mn^2+^ have different orientations within the metal-binding site due to cation preference to a given type of bond in RNA. Thus, the position of Mg^2+^ being a solid ion strongly depends on the tight coupling of P–O, while Mn^2+^ is closer to the N7 position of guanine, wherein the interaction with the oxygen of the previous phosphodiester bond is maintained [31]. Transition metals bind nucleic acid more tightly than Mg^2+^ or K^+^, which is largely due to the interaction with nucleophilic nitrogenous bases (N7 purine) [17].

Therefore, the characteristic behaviour of Mn^2+^ in the NMR spectroscopy of RNA can be explained by its affinity to N7 purines (Figure 2(d)). Guanine binding of Mn^2+^ ions either limits accessibility for guanyl-specific binase sites in RNA or hinders the recognition of guanine. As a result, we observed no increase in the rate of 2′,3′-cGMP, as a reaction intermediate catalyzed by cyclizing RNases (Figure 3(b)). Based on the obtained evidence on the ability of nontransition metal ions to activate 2′,3′-cGMP, which is not the case of transition metal ions, it can be assumed that the investigated transition metals affect RNA-binase binding sites; the related impact is particularly strong in those cases where RNA is stabilized by phosphate groups.

Binase is a promising therapeutic agent with anticancer [32–36] and antivirus effects [37, 38]. Secretion of RNase by bacilli in the intestinal flora is a natural defense mechanism that counters oncogenesis and viral infections. The activation of 2′,3′-cGMP by nontransition metals in the human body can definitely support biological effects of microbial RNases.

## 5. Conclusions

It was shown that the rate of 2′,3′-cGMP, produced by binase during RNA cleavage, was increased in the presence of nontransition metals that stabilized the RNA structure. In the case of transition metal ions the amount of 2′,3′-cGMP was reduced because of blocked guanine in binase recognition sites of RNA. As production of RNase by bacilli in the human body is a natural mechanism, the activation of 2′,3′-cGMP by nontransition metals can enhance biological effects of binase.
